# The Role of Metal–Organic Framework Induced Confinement Effects on Molecular Electrocatalysts Relevant to the Energy Transition

**DOI:** 10.1002/cssc.202402676

**Published:** 2025-04-24

**Authors:** Marlene E. Hoefnagel, Dennis G. H. Hetterscheid

**Affiliations:** ^1^ Leiden Institute of Chemistry Leiden University Einsteinweg 55 2333 CC Leiden The Netherlands

**Keywords:** confinement effects, electrocatalysis, electron transport, mass transport, metal organic frameworks

## Abstract

Metal organic frameworks (MOFs) are promising materials for (electro)catalysis as they can improve stability, reusability, and catalytic current densities of molecular catalysts, thereby combining the advantages of homogeneous‐ and heterogeneous catalysts. However, much is unknown about the effects of confinement of a catalyst within an MOF on the overall catalytic behavior. The performance of a series of electrocatalysts confined in MOFs is compared to that of the corresponding homogeneous catalysts to evaluate to what extend the catalytic site is affected by confinement in terms of stability, activity, and selectivity. Together the examples discuss depict what happens to a catalyst when it is incorporated into an MOF, and recommendations are made on how to evaluate the electrochemical activity of an MOF in a way that allows for description of such confinement effects on the catalyst performance. It is noted that the limiting factor for the catalytic reaction in MOFs is found in 1) slow electron transport, 2) slow mass transport of reactants and products, or 3) a low activity of the catalytic site itself. Understanding the changes in mass‐ and electron transport and the resulting effects on catalytic mechanism is essential to be able to bring MOF systems to practical applications.

## Graphical abstract

In this review, the performance of a series of electrocatalysts confined in MOFs is compared to that of the corresponding homogeneous catalysts to evaluate to what extend the catalytic site is affected by confinement in terms of stability, activity and selectivity. Together, the examples discussed depict what happens to a catalyst when it is incorporated into an MOF.

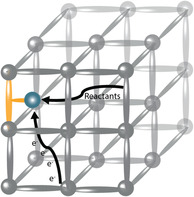

## 1 Introduction

The climate crisis is largely caused by the combustion of fossil fuels to meet energy demands. Combustion of fossil fuels releases greenhouse gasses that result in an increase of temperature, melting of land‐ and sea ice, ocean acidification, loss of biodiversity, draughts, forest fires, and more.^[^ ^1^, ^2^ ^]^ The effects of climate change hit hardest to those in underdeveloped regions, further increasing their vulnerability. Simultaneously, the number of people with access to the energy market is rapidly increasing and therefore so is the amount of energy needed.^[^ ^3^ ^]^ With 16200 TW per year, the sun provides an abundant amount of energy to meet all our demands.^[^ ^4^ ^]^ However, the intermittent availability of sunlight gives rise to the need to store the energy obtained from solar cells. Solar energy is converted to chemical energy by the synthesis of fuels. Production of fuels can either proceed directly by photochemical approaches or sequentially by photovoltaics and electrolysis. The electricity generated by photovoltaics may also be stored in batteries, albeit the energy density of most battery systems is low.^[^ ^5^ ^]^ Suitable fuels consist of small molecules such as hydrogen that is produced by electrochemical water splitting, and carbon‐based fuels that is obtained by CO_2_ reduction.^[^ ^6^ ^]^ Electrocatalysis is expected to play a significant role in the shift from a fossil fuel‐based to a more renewable energy economy.^[^ ^7^, ^8^ ^]^ The most common electrocatalytic reactions that are relevant for renewable energies are hydrogen evolution (Equation (1)), water oxidation (Equation (2)), carbon dioxide reduction (Equation (3) and (4)), and oxygen reduction (Equation (5) and (6)).

(1) $${\text{4H}}^{+}+{\text{4e}}^{-}\to H_{2}\;\text{E°}=0\;V$$

(2) $${\text{2 H}}_{2}\text{O }\to O_{2}+{\text{4H}}^{+}+{\text{4e}}^{-}\;\text{E°}=\text{1.23 V}$$

(3) CO2+2H++2e−→CO+H2O   E°=−0.52 V

(4) $${\text{CO}}_{2}+H^{+}+{\text{2e}}^{-}\to {\text{HCOO}}^{-}\;\;\text{E°}=-0.61\text{ V}$$

(5) $$O_{2}+{\text{4H}}^{+}+{\text{4e}}^{-}\to {\text{2 H}}_{2}O\;\text{E°}=\text{1.23 V}$$

(6) $$O_{2}+{\text{2H}}^{+}+{\text{2e}}^{-}\to {\text{2 H}}_{2}O_{2}\;\text{E°}=\text{0.7 V}$$

To successfully transition to a renewable energy economy, development of better catalysts for the reactions above is indispensable. New and emerging technologies and strategies may be essential as well. Electrocatalysts can be either heterogeneous, such as materials and surfaces mostly based on metals, or homogeneous, such as coordination complexes of transition metals. Heterogeneous catalysts have the advantage of a large number of active sites at the electrode surface, allowing for large catalytic currents and high product yields.^[^ ^9^ ^]^ However, catalytic mechanisms and the true identity of catalytically active species are often ambiguous, making optimization of material properties difficult. Homogeneous catalysts have the advantage of precise tunability of their structure and therefore allow for studying and utilizing structure‐activity relationships. However, homogeneous catalysts often suffer from instability, poor recoverability, poor scalability, and are difficult to recycle.^[^ ^10^ ^]^ Immobilization of homogeneous electrocatalysts onto electrode surfaces can help overcome these problems and combine some of the advantages of both heterogeneous and homogeneous electrocatalysts.^[^ ^11^ ^]^ Particularly the incorporation of molecular catalysts in metal–organic frameworks (MOFs) has received a lot of attention lately, due to potential that these systems have shown thus far for catalytic purposes.

### 1.1 MOFs as Electrocatalysts

MOFs are highly symmetrical 3D coordination polymers that consist of metal nodes and organic linkers. The high porosity of these systems results in high catalytic surface areas and the possibility to vary the structure of the framework infinitely by alterations of the linkers and nodes, which make them interesting materials to tune the (electronic) structure of the catalyst in the first‐, second‐, and even third‐coordination spheres.^[^ ^12^, ^13^, ^14^, ^15^ ^]^ This is essential given that precise tuning of the catalyst favorable to the specific reaction that they catalyze is essential.^[^ ^16^ ^]^ Moreover, incorporation of catalysts into an MOF has been shown to increase the stability and reusability of the catalytic site in some occasions, as well as influence selectivity and rates of the catalytic reaction by confinement effects in other examples.^[^ ^17^, ^18^, ^19^ ^]^ Simultaneously, the number of active sites per unit of surface area can be pushed to numbers far beyond that of typical heterogeneous catalysts, while bimolecular reactions leading to undesired side phenomena can be rigorously shut down.

**Figure 1:**
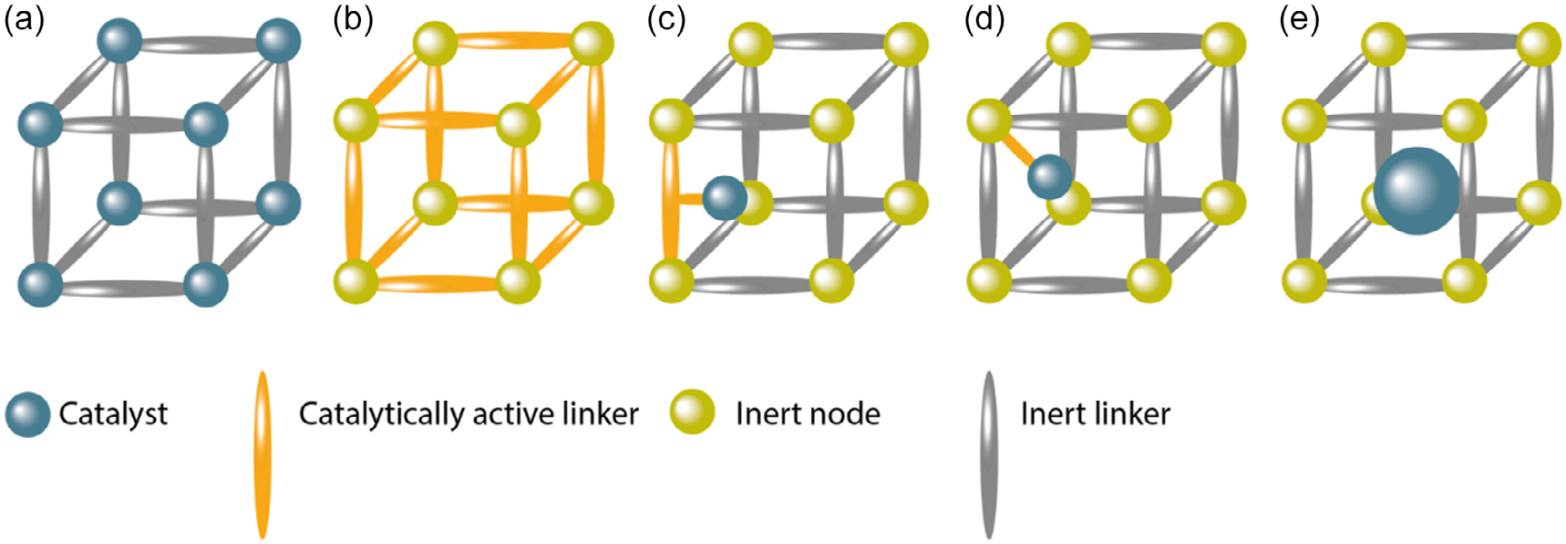
Different strategies to produce catalytically active MOFs are shown: a) the node itself is catalytically active; b) the linker is the catalyst; c) the catalyst is anchored to the linker during the synthesis or through post synthetic ligand exchange strategies; d) the catalyst is bound to the node via solvent assisted ligand incorporation; e) the catalyst is trapped in the pores via the ship‐in‐a‐bottle approach.

Molecular catalysts can be incorporated into MOFs by various strategies, as summarized schematically in **Figure** **1**:^[^ ^20^ ^]^ 1) An MOF can contain catalytically active nodes (Figure 1a) such as for example the MIL‐100 framework, wherein the Sc^3+^/Fe^3+^ nodes are active for tandem C—C bond formation and alcohol oxidation and^[^ ^21^ ^]^ 2) the catalytic site can be imbedded in the linker (Figure 1b), of which porphyrinic PCN frameworks are a good example. In these frameworks, four carboxylic acids moieties on the porphyrin bind to the Zr‐cluster nodes;^[^ ^22^, ^23^ ^]^ 3) A catalyst can be trapped inside MOF pores during synthesis by the “ship‐in‐a‐bottle” approach (Figure 1e);^[^ ^24^ ^]^ 4) Homogeneous catalysts can be introduced into the MOF by post‐synthetic modification (PSM) methods. An example of PSM is post synthetic ligand exchange (Figure 1c), as reported for the [FeFe]‐hydrogenase functionalized UiO‐66 framework. Here some of the dicarboxybenzene ligands are replaced by dicarboxybenzene ligands functionalized with the [Fe—Fe]‐hydrogenase catalysts a, by simply stirring a solution containing the MOF in presence of the catalyst.^[^ ^25^ ^]^ Another form of PSM is solvent‐assisted ligand exchange (SALI) (Figure 1d), where the MOF is soaked in a concentrated solution of catalyst at elevated temperatures. Within this strategy, for example, hydroxyl groups on MOF nodes are replaced by carboxylic acid moieties on the catalyst by soaking the MOF in a concentrated solution of the carboxylic acid containing molecular catalyst.^[^ ^26^, ^27^ ^]^

Various methods to mount the MOFs onto electrodes have been discussed in the literature. The most common method is to drop cast an ink containing the MOF, a conductive carbon support such as carbon black or multiwalled carbon nanotubes (MWCNTs), and a binder such as Nafion or Sustainion.^[^ ^22^, ^27^, ^28^, ^29^ ^]^ Alternatively, MOFs can be grown solvothermically onto FTO or ITO electrodes.^[^ ^30^, ^31^, ^32^ ^]^

When a catalyst is incorporated into an MOF, the aforementioned confinement effects are expected to affect the mechanism and rate‐determining step of the catalytic reaction to some extent. Due to such confinement effects, for a given electrocatalytic process, the limiting factor for the overall reaction may be found in slow electron transport between the active sites and the electrode; a limited mass transport of reactants and products; or be limited by the intrinsic activity of the catalytic site itself. Electron transport is expected to be the limiting factor when the redox‐active moieties are diffuse, and charge propagation is expected to depend on electron hopping from one catalytic center to another. In this case, the catalytic reaction will likely take place at the electrode surface, where electrons are injected into the MOF. Alternatively, when mass transport of reactants and products is rate limiting, the catalytic reaction is expected to take place at the MOF—electrolyte interface where new reactants are replenished first. Lastly, if the catalytic reaction is limited by the activity of the catalyst itself, catalysis is expected to take place throughout the entire MOF. Since the mass‐ and charge transport mechanisms within the MOF are undoubtedly different from mass and charge transport mechanisms in the homogeneous situation, a comparison between the confined catalyst and the catalyst in homogeneous solution may be very insightful. Performance of catalysts is often evaluated by turnover numbers (TONs), which represent the stability of a catalyst, and turnover frequencies (TOFs), which represent the activity of the catalyst.^[^ ^33^ ^]^ It is however important to note that TONs are difficult to determine for homogeneous electrocatalysts, as it requires determination of the exact number of catalytic species present at the electrode surface. This is far from straightforward to determine due to the diffusive nature of the catalytic species, which makes drawing conclusions in terms of improved stability through confinement often problematic. Nevertheless, understanding how confinement effects affect the catalytic activity, mass, and electron transport throughout MOFs is essential to be able to bring such MOF systems to practical applications.

Various comprehensive reviews have already discussed the use of MOFs as electrocatalysts,^[^ ^14^ ^]^ how to evaluate the electrochemical performance of these MOFs,^[^ ^34^ ^]^ redox active MOFs for energy storage and conversion,^[^ ^35^ ^]^ and coordination sphere effects on MOF electrocatalysts.^[^ ^13^ ^]^ However, a review that focuses on the effect of confinement on the catalyst within the MOF, and how confinement changes the overall catalytic behavior of the active site has thus far not been reported. In this review, we discuss a selection of studies on molecular electrocatalysts immobilized in metal organic frameworks for catalytic reactions relevant to the energy transition. We critically compare the performance of the electrocatalysts confined in MOFs to that of the parent homogeneous catalyst to evaluate to what extend the catalytic site is affected upon confinement in terms of stability, activity, and selectivity. Together, the examples discussed paint an overall picture of what happens to a catalyst when it is incorporated into an MOF, and recommendations are made on how to evaluate the electrochemical activity of an MOF in a way that allows for an accurate description of such confinement effects on the observed catalysts performance.

## 2 Discussion

### 2.1 Hydrogen Evolution

#### 2.1.1 Cobaloxime

**Figure 2:**
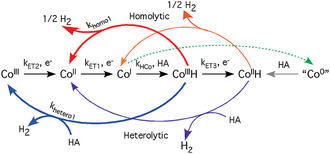
Schematic pathway of the most common pathways resulting in H_2_ evolution catalyzed by cobaloximes. Reproduced with permission.^[^ ^36^ ^]^ 2009, American Chemical Society.

Cobalt complexes with diglyoxime ligands, also known as cobaloximes, are among the most intensively investigated homogeneous catalysts for the hydrogen evolution reaction (HER).^[^ ^36^ ^]^ Within the catalytic reaction, a Co^III^ cobaloxime is reduced to a Co^II^ or Co^I^ species that is protonated to form a Co^II^‐hydride or a Co^III^‐hydride. Here the precise details depend on the electronic structure of the complex. Irrespective whether a Co^III^—H or Co^II^—H cycle is formed, formation of the active hydride is typically the rate‐determining step. The catalytic reaction can proceed via four different pathways, either by a homolytic‐ or a heterolytic H—H bond formation, and either occurring at the Co^II^—H or a Co^III^—H species, as schematically shown in **Figure** **2**. In the homolytic pathway, two Co‐hydride species react in a bimolecular reaction to release hydrogen, while in the heterolytic pathway the Co‐hydride is protonated to produce H_2_ and Co^III/II^. The homolytic pathway proceeds via intermediates that can be generated at milder potentials and depends on the diffusion of two cobalt hydride species toward each other in solution. However, a reaction between two such species can also trigger a catalyst degradation trajectory that occurs via a bimolecular deactivation pathway.^[^ ^37^, ^38^ ^]^ Overreduction of the cobaloxime was found to generate carbon‐based α‐imino radical species that react with each other to form inactive dimers. Therefore, directing the catalytic mechanism toward the heterolytic HER pathway by immobilization of the cobaloxime is anticipated to improve the stability of the catalyst, albeit with a larger overpotential as a direct consequence. In addition to this bimolecular degradation pathway, the instability of cobaloximes may be caused by hydrogenation of the ligand triggered by a hydride migration from the Co^I^LH species. This degradation pathway can be prevented by direct reduction of this Co^I^LH species to the catalytically competent Co^II^—HLH species.^[^ ^37^ ^]^ Immobilization of the catalyst on an electrode allows for rapid reduction reactions and may prevent dimerization and can therefore slow down cobaloxime degradation, explaining the improved stability of immobilized cobaloxime compared to homogenous equivalents. Following such a strategy, cobaloximes have been immobilized on, amongst others, metal oxides, carbon nanotubes (CNT), liposomes, and MOFs.^[^ ^39^, ^40^, ^41^, ^42^ ^]^

**Figure 3:**
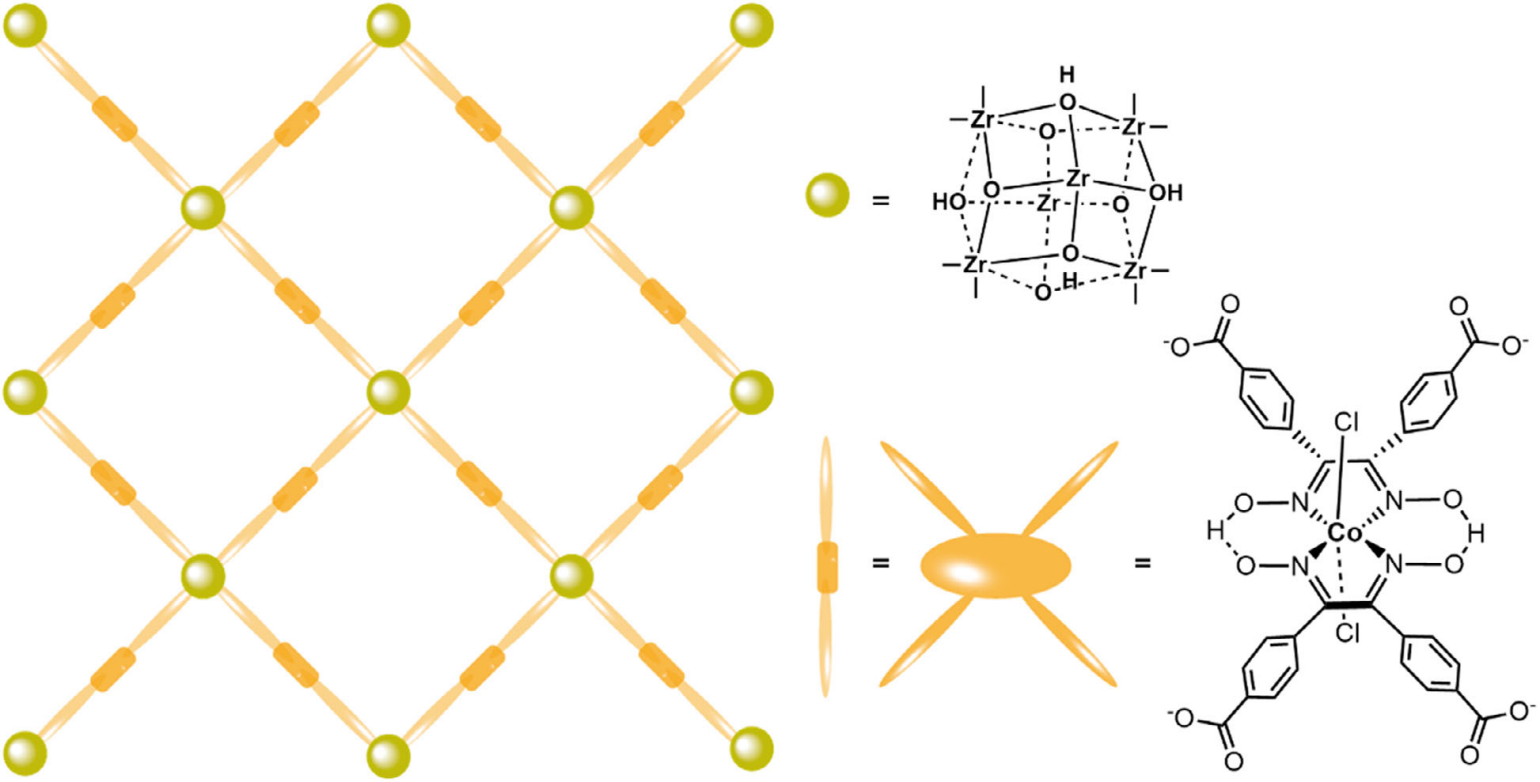
Schematic presentation of the **UU‐100(Co)** MOF.

In the MOF approach, a cobaloxime was equipped with four benzenecarboxylic acid moieties and used as a linker in the **UU‐100(Co)** MOF with hexanuclear zirconium nodes (**Figure** **3**) and studied as a catalyst for the HER.^[^ ^32^ ^]^ The cobaloximes were found to promote electron transfer throughout the film, by UV‐vis spectroelectrochemistry and Cottrell analysis, as well as function as molecular hydrogen evolution catalysts. High catalytic current densities were obtained during chronoamperometry at −0.45 V vs. RHE for 18 h at pH 4, with an FE around 80% for H_2_. Keeping in mind the discrepancies between homogeneous and heterogeneous TONs, incorporation of the cobaloxime into the framework greatly increased its stability (**Table** **1**). The turnover number went up from 10 for the linker in homogeneous solution to more than 20000 when imbedded within the MOF. This was attributed to the rigid 3D structure of the MOF that rigorously prevents dimer formation. The stability of the MOF also compares favorably to that of cobaloximes immobilized on CNTs and composites of CNTs and polymers, for which TONs between 120 and 420 were obtained.^[^ ^40^, ^41^ ^]^ Formation of cobaloxime dimers in the rigid framework of the MOF is even more difficult than when grafted onto carbon substrates and may contribute to the large improvement in stability in the MOF embedded catalyst compared with other immobilization methods. Cobaloximes are not stable at low pH (<2.2) and complete dissociation leading to metallic depositions on the electrode have been reported.^[^ ^43^ ^]^ Little is known regarding local pH effects within MOFs, and to which extend alkalization within the MOF pores contributes in some extend to its stability during catalysis.^[^ ^44^, ^45^ ^]^

**Table 1:** TONs obtained for cobaloxime‐based hydrogen evolution systems.

Species	TON [s^−1^]
Cobaloxime linker (hom)a)	10^[^ ^32^ ^]^
UU‐100(Co)b)	>20 000^[^ ^32^ ^]^
Cobaloxime on CNTc)	120^[^ ^40^ ^]^
Cobaloxime‐polymer composit on CNTd)	420^[^ ^41^ ^]^

a) −0.45 V vs. RHE, acetate buffer pH 4, WE = carbon rod;

b) −0.43 V vs. RHE, acetate buffer pH 5.3, WE = FTO;

c) −0.33 7 V vs. RHE, NaCl pH 7, WE = glassy carbon;

d) −0.045 V vs. RHE, phosphate buffer, pH 7, WE = CNT buckypaper.

### 2.2 CO_2_ Reduction

#### 2.2.1 Co‐Phtalocyanine

Co‐phtalocyanines (CoPCs) are among the most investigated and most stable homogeneous catalysts for the reduction of CO_2_ to CO.^[^ ^46^ ^]^ The mechanism of CO_2_ reduction proceeds by reduction of Co^II^ to Co^I^, binding of CO_2_ and transfer of an electron from Co to CO_2_. This electron transfer from Co to CO_2_ is the rate‐determining step and is coupled to transfer of a proton at high carbonate concentrations or followed by a separate proton transfer step at low carbonate concentrations.^[^ ^47^ ^]^ In electrocatalytic CO_2_ reduction by CoPC, the true catalytically active species is not homogeneous, but an electro‐absorbed CoPC species on the electrode. Aromatic macrocycles such as phtalocyanine tend to aggregate,^[^ ^47^ ^]^ leading to inactive oligomeric stacks. As a result, a water‐soluble CoPC studied by Wu et al. in aqueous electrolyte lost more than 50% of its activity within 30 min due to stacking.^[^ ^48^ ^]^ However, Warren and coworkers recently reported a water‐soluble Co‐phtalocyanine with cationic groups that showed minimal aggregation and high turnover frequencies.^[^ ^49^ ^]^ Controlled potential electrolysis (CPE) experiments were only performed for 20 min, making it difficult to comment on any long term stability. To maximize the number of active species at the electrode interface, CoPCs were immobilized on carbon‐based scaffolds^[^ ^50^ ^]^ and gas diffusion electrodes (GDEs).^[^ ^51^ ^]^ Interestingly, immobilization of amine‐substituted CoPC on CNTs enables formation of methanol as the product.^[^ ^52^ ^]^ Immobilization of CoPC on GDEs results in high current densities for the reduction of CO_2_ to CO, (FE_CO_ = 95%) at a catalytic current that is stable for at least 12 h, indicating the improved catalytic stability of the immobilized Co‐phtalocyanine compared to homogeneous Co‐phtalocyanine.

**Figure 4:**
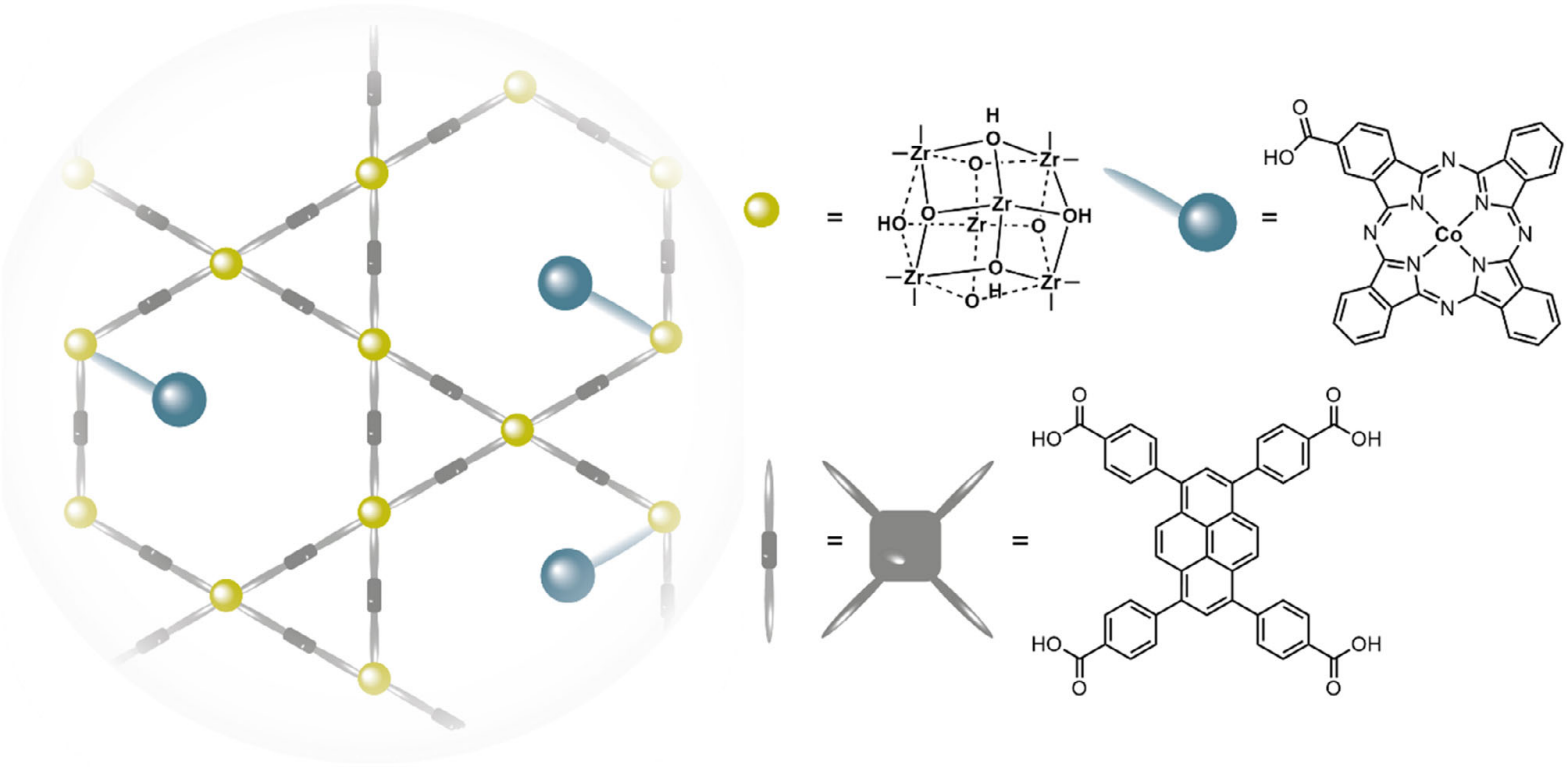
Schematic presentation of the **NU1000|CoPC** MOF.

Co‐phtalyocyanine was immobilized in the Zr‐based NU1000 MOF by attaching the catalyst to the MOF nodes by solvent assisted ligand incorporation to form **NU1000|CoPC** (**Figure** **4**).^[^ ^26^ ^]^ In order to investigate to which extend electron transfer between the CoPC catalyst and the NU1000 linker is of influence for catalytic performance, a Co‐porphyrin was also incorporated into NU1000 to form **NU1000|CoPor** via the same procedure. The observed superior activity of the **NU1000|CoPC** MOF compared with the **NU1000|CoPor** MOF was attributed to reduced NU1000 being capable of reducing cobalt to generate the key Co^0^ species, whereas a larger overpotential is necessary to reduce Co(TPP) to the same oxidation state. The **NU1000|CoPC** MOF formed CO with faradaic efficiencies ranging from 40–70% for applied potentials between −0.55 and −0.85 V vs. RHE (**Table** **2**). In case of **NU1000|CoPC** substantially more hydrogen was produced compared to CoPC immobilized on a GDE, which showed FE_CO_ = 95%. We anticipate this difference in faradaic efficiency for CO could be caused by slow mass transport of CO_2_ in the MOF pores compared to a homogeneous system. As mass transport of CO_2_ consumed during CO2RR in MOF pores is significantly more difficult than on a GDE, where a constant flow is maintained, the lower FE_CO_ for the MOF‐immobilized CoPC could be explained by a less rapid flow of CO_2_ to the catalytic sites. On the other hand, it is important to note that reduced mass transport can also result in a higher local pH, as protons are consumed during the CO2RR, which would then result in a higher FE_CO_. Not only selectivity for CO, but also catalytic currents decreased over time in case of the **NU1000|CoPC** MOF. Chronoamperometry at –0.65 V vs. RHE resulted in a current of 1.75 mA cm^−2^ that decreased ≈30% over 4 h. In agreement with this the current densities decreased over consecutive CV scans. These observations indicate catalyst instability. As immobilization of the phtalocyanine in the MOF pore prevents aggregation, degradation of the CoPC active sites must proceed via a different mechanism than was described for homogeneous phtalocyanines. An MOF system with a Co‐phtalocyanine equipped with four carboxylic acid groups as the linker and Fe‐based nodes (**MOF‐1992**) showed a good faradaic efficiency (FE_CO_ = 80%) and a stable catalytic current for at least two hours.^[^ ^53^ ^]^ Both **MOF‐1992** and **NU1000|CoPC** showed lower FE for CO as the product compared to the homogeneous phtalocyanine. Additionally, a local increase of pH in the MOF pores might cause the bond between the Zr‐node and the CoPC to be hydrolyzed, resulting in detachment and removal of the CoPC species from the MOF.

**Table 2:** Farradaic efficiency for CO for phtalocyanine‐based systems.

Species	E [V vs. RHE]	pH	FE_CO_ [%]
CoPC on GDEa)	−0.72	7.3	96^[^ ^51^ ^]^
NU1000|CoPCb)	−0.85	8.5	44^[^ ^26^ ^]^
NU1000|CoPCb)	−0.75	8.5	56^[^ ^26^ ^]^
NU1000|CoPCb)	−0.65	8.5	75^[^ ^26^ ^]^
NU1000|CoPCb)	−0.55	8.5	55^[^ ^26^ ^]^
MOF‐1992c)	−0.63	6.8	80^[^ ^53^ ^]^

a) NaHCO_3_, WE = glassy carbon;

b) KHCO_3_, WE = graphite sheet;

c) KHCO_3_, WE = glassy carbon.

### 2.3 Re(bpy)(CO)_3_

Re(bpy)(CO)_3_ was first reported in 1984 by Lehn and has been investigated for the CO_2_ reduction reaction ever since.^[^ ^54^, ^55^ ^]^ High catalytic rates and selectivity to form CO have been reported, but instability of the catalyst, caused by dimerization, has been a recurring problem.^[^ ^56^ ^]^ With this in mind, immobilization of the catalyst in a manner that prevents dimerization reactions may result in a higher catalyst stability. Various ways of immobilizing Lehn's catalyst have been explored including immobilization in liposomes, on mesoporous organosilica and on carbon electrodes as a polymer thin film.^[^ ^57^, ^58^, ^59^ ^]^

**Figure 5:**
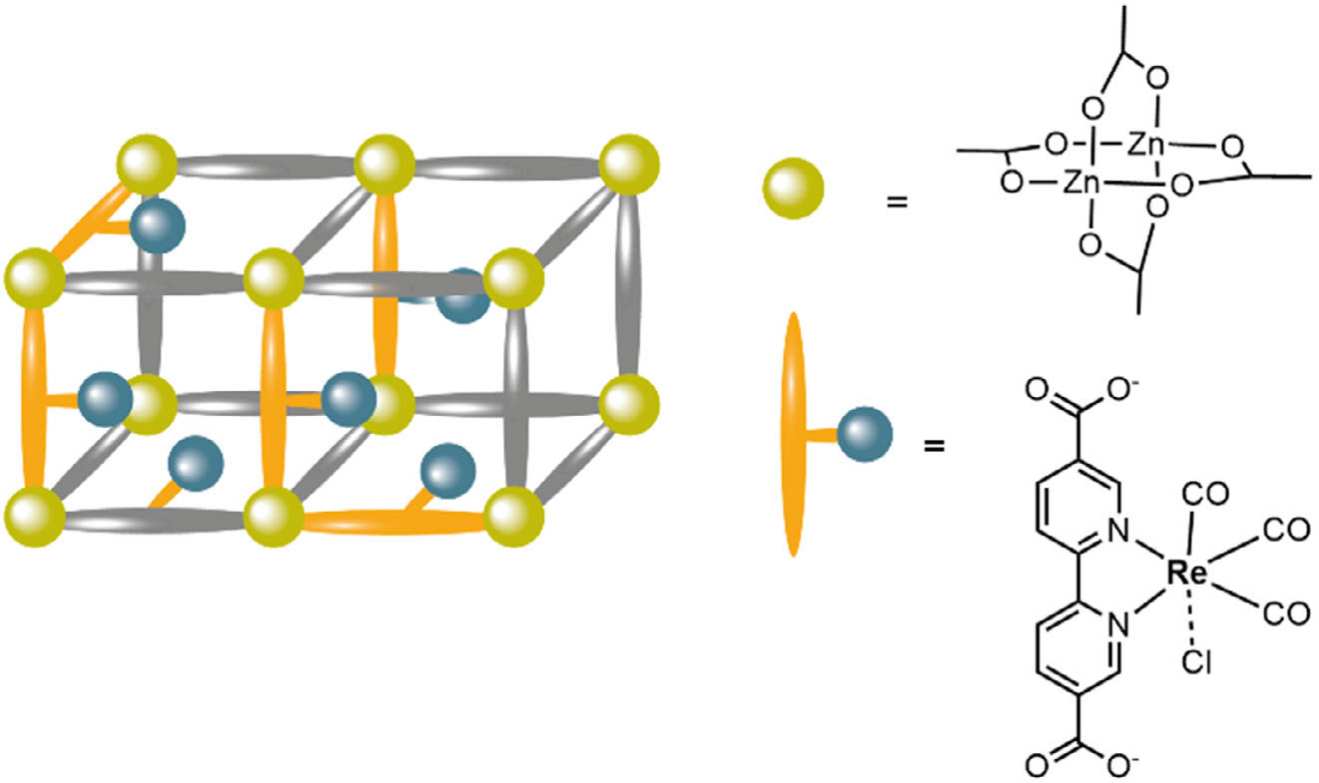
Schematic presentation of the structure of **Re‐SURMOF**.

Re(bpy‐dicarboxylic acid)(CO)_3_ catalysts were incorporated as linkers into the Zn‐based MOF thin film **Re‐SURMOF** (**Figure** **5**) by lipid phase epitaxy onto an FTO substrate.^[^ ^60^ ^]^ The MOF was found to reduce CO_2_ to CO with a high faradaic efficiency of 93% for chronoamperometry at −1.6 V vs. NHE in ethanol (**Table** **3**). The MOF film was compared to both Re(bpy‐dicarboxylic acid)(CO)_3_ in homogeneous solution as well as its dropcasted form onto an FTO electrode, for which lower faradaic efficiencies to CO were found with 65% for the homogeneous linker and 61% for the dropcasted linker. The **Re‐SURFMOF** showed higher catalytic current densities as well, which the authors attribute to the highly oriented structure of the MOF film to the electrode surface. This allows for efficient charge transport along the [001] direction of the framework, which ensures activation of catalytic sites further from the electrode surface. In contrast to the MOF system, in homogeneous solution only a limited number of catalysts are close enough to the electrode surface to be activated, and a dropcast of catalysts may be too amorphous to ensure sufficient contact with electrode and electrolyte. However, the MOF thin film showed to be instable when CA experiments were continued for more than 30 min, and all catalytic activity was lost within 2 h of electrolysis. As dimerization reactions seem unlikely within the MOF, the catalyst degradation reactions must proceed via a different mechanism than in homogeneous solution. XRD of the electrode after electrolysis confirmed that delamination of the MOF thin film has taken place. Possibly, the bonds between the carboxylic acid moieties attached to the catalyst and Zn and/or FTO are not stable and hydrolyze, resulting in the entire film dissolving.^[^ ^58^ ^]^

**Table 3:** Farradaic efficiency for CO for Re(bpy)(CO)_3_‐based systems.

Species	*E* [V vs. NHE]	FE_CO_ [%]
Re‐SURMOF	–1.6a)	93^[^ ^60^ ^]^
Re(bpy)(CO)_3_ homogeneous	–1.6	65^[^ ^60^ ^]^
Re(bpy)(CO)_3_ dropcasted	–1.6	61^[^ ^60^ ^]^

a) TBAH + 5% trifluoroethanol in acetonitrile, WE = FTO.

## 3 Oxygen Reduction

### 3.1 Co‐Porphyrin

Various Co‐porphyrins that can catalyze the oxygen reduction reaction (ORR) at a relatively low overpotential have been designed to direct the selectivity of ORR toward either water or hydrogen peroxide by adding steric groups as well as electronic effects on the porphyrin ring.^[^ ^61^, ^62^, ^63^, ^64^, ^65^ ^]^ Aromatic macrocycles such as porphyrins are often best described as heterogeneous catalysts as they tend to aggregate in aqueous solution, which makes them stick to the hydrophobic surface of electrode materials such as glassy carbon.^[^ ^47^, ^61^ ^]^ Therefore, its especially important for this type of catalysts to determine the nature of the catalytically active species.

**Figure 6:**
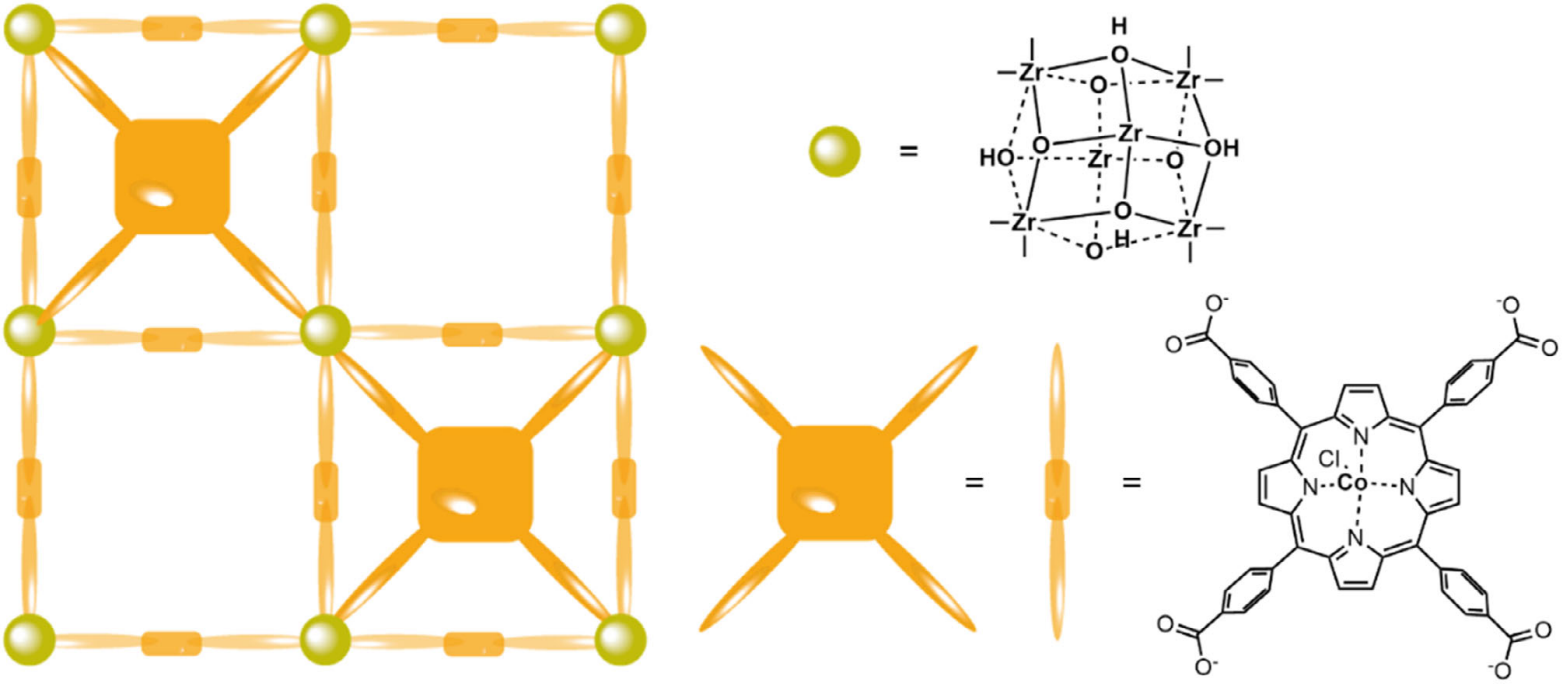
Schematic presentation of the structure of **PCN‐224(Co).**

Co‐porphyrin was applied as a linker in the **PCN‐224(Co)** MOF (**Figure** **6**) to catalyze the ORR toward hydrogen peroxide.^[^ ^28^ ^]^ The MOF was compared with the Co‐porphyrin containing linker dropcasted onto an electrode and showed an improved FE for hydrogen peroxide of 80%, compared to 30% for the free linker, albeit with a somewhat decreased current density. Dimers of Co‐porphyrin are reported to favor H_2_O as the main product, while monomeric catalytic sites often favor the formation of H_2_O_2_. Therefore, the improved FE was ascribed to immobilization of the catalyst in the ordered framework of the MOF, thereby preventing dimerization of the catalytic species. Fully in line with this hypothesis is the observation that aggregation of Co‐macrocycle catalysts in solution was found to decrease the selectivity and result in production of only small amounts of H_2_O_2_ product.^[^ ^47^ ^]^

### 3.2 Cu‐Tmpa

With almost 10^6^ turnovers per second, Cu‐tmpa is one of the fastest catalysts for the ORR reported thus far.^[^ ^66^ ^]^ The catalytic mechanism proceeds via reduction of the Cu^II^ to the Cu^I^ species and subsequent binding of dioxygen, which is the rate‐determining step. Hydrogen peroxide is formed as an intermediate, which can be further reduced to water in the hydrogen peroxide reduction reaction (HPRR).^[^ ^67^ ^]^ The high TOF is attributed to the electron donating properties and flexibility of the tetradentate ligand, which allows for tetrahedral coordination upon reduction, which is the preferred coordination geometry of Cu^I^ species.^[^ ^68^ ^]^ Direct immobilization of Cu‐tmpa as a self‐assembled monolayer onto a gold electrode has resulted in formation of inactive clusters.^[^ ^69^ ^]^

**Figure 7:**
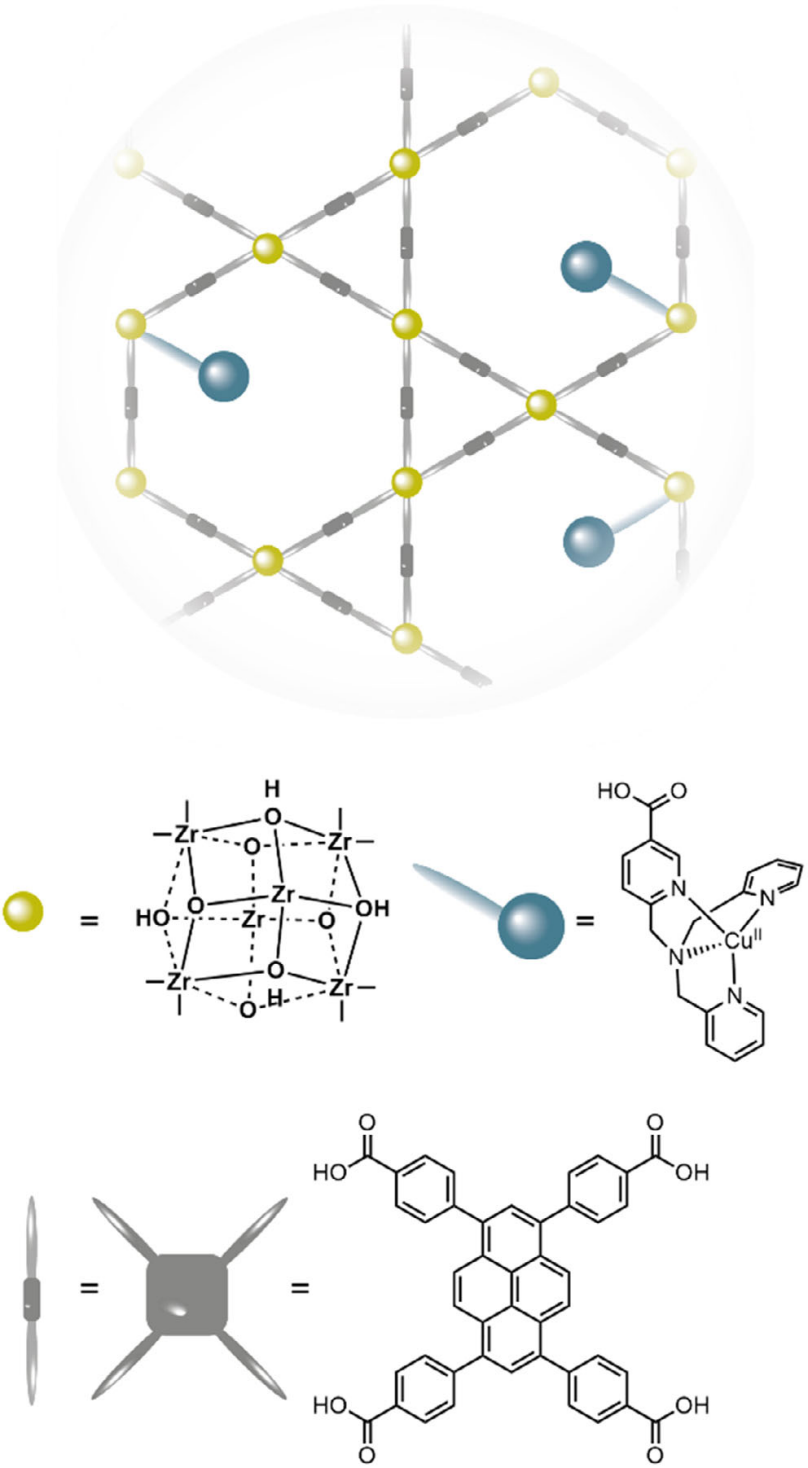
Schematic presentation of the structure of **NU1000|Cu‐tmpaCOOH**.

Cu‐tmpaCOOH was immobilized in the pores of NU1000 MOF by binding of the catalyst to the Zr‐nodes via SALI (**Figure** **7**) and studied for the oxygen reduction reaction.^[^ ^27^ ^]^ The Cu‐tmpaCOOH‐functionalized MOF was directly compared to the Cu‐tmpaCOOH catalyst in homogeneous solution and showed an improved catalytic current density (**Table** **4**), high stability, and reusability. The analogous Cu‐tmpa catalyst was reported to reach catalytic current densities of 3.2 mA cm^−2^ for a 0.3 mM catalyst concentration and 1 mA cm^−2^ for a 5 μM catalyst concentration, while the **NU1000|Cu‐tmpaCOOH** MOF showed a catalytic current density of 3.5 mA cm^−2^ for 96 nmol Cu in the MOF on the electrode.^[^ ^70^ ^]^ The homogeneous Cu‐tmpa showed a current decrease from –0.4 to –0.2 mA over 28 h CPE measured in three days with significant deposition of metallic Cu on the electrode. On the contrary, the **NU1000|Cu‐tmpaCOOH** MOF showed minimal decrease in current during 30 h CPE. A shift in selectivity from H_2_O_2_ to H_2_O as the preferred product was observed upon immobilization of Cu‐tmpaCOOH into the framework. An interesting observation is that post catalysis a second redox couple is present in the voltammetry of **NU1000|Cu‐tmpaCOOH**, which may point to the presence of copper particles.^[^ ^71^ ^]^ Cu‐clusters were generated separately within NU1000 by treatment of NU1000 with Cu(OTf)_2_. Although, given that **NU1000|Cu(OTf)**_**2**_ is inactive, the presence of Cu‐tmpaCOOH must be essential to obtain catalytic activity and may be explained in terms of Cu‐tmpaCOOH being relevant for electron transfer rather than being the exclusive catalytic site. Additionally, the retention of H_2_O_2_ within the MOF pores and therefore low probability to escape from the active site may be an explanation for the shift in selectivity compared to the homogeneous system.^[^ ^72^ ^]^

**Table 4:** ORR current densities of Cu‐tmpa‐based systems.

Species	Cu‐concentration	I [mA cm^−2^]
Cu‐tmpa	0.3 mMa)	3.2^[^ ^70^ ^]^
Cu‐tmpa	0.005 mM	1^[^ ^70^ ^]^
NU1000|Cu‐tmpaCOOH	96 nmol	3.5^[^ ^27^ ^]^

a) Phosphate buffer pH 7, WE = glassy carbon, 0.3 V vs. RHE.

## 4 Water Oxidation

### 4.1 Ru(tpy)(dcbpy)OH_2_

The redox properties of [Ru(tpy)(bpy)(OH_2_)], (tpy = terpyridine, bpy = bipyridine) were characterized by Meyer and coworkers as early as 1983,^[^ ^73^ ^]^ and they later also started investigating the complex as a catalyst for water oxidation reaction.^[^ ^74^, ^75^ ^]^ Formation of the O—O bond at Ru water oxidation catalysts (WOCs) is typically the rate‐determining step^[^ ^76^ ^]^ and can proceed via an interaction between two M—O species (I2M) resulting in direct formation of O_2_, or via a water nucleophilic attack (WNA) mechanism.^[^ ^77^ ^]^ In the WNA mechanism, the O—O bond is formed by nucleophilic attack of water on a high‐oxidation state M=O species. In most cases, proton transfer plays a role in the WNA mechanism, for which high solvent kinetic isotope effects and buffer concentration dependencies are good diagnostic tools.^[^ ^78^ ^]^ [Ru(tpy)(bpy)(OH_2_)] catalysts perform water oxidation via the WNA mechanism.^[^ ^75^ ^]^

**Figure 8:**
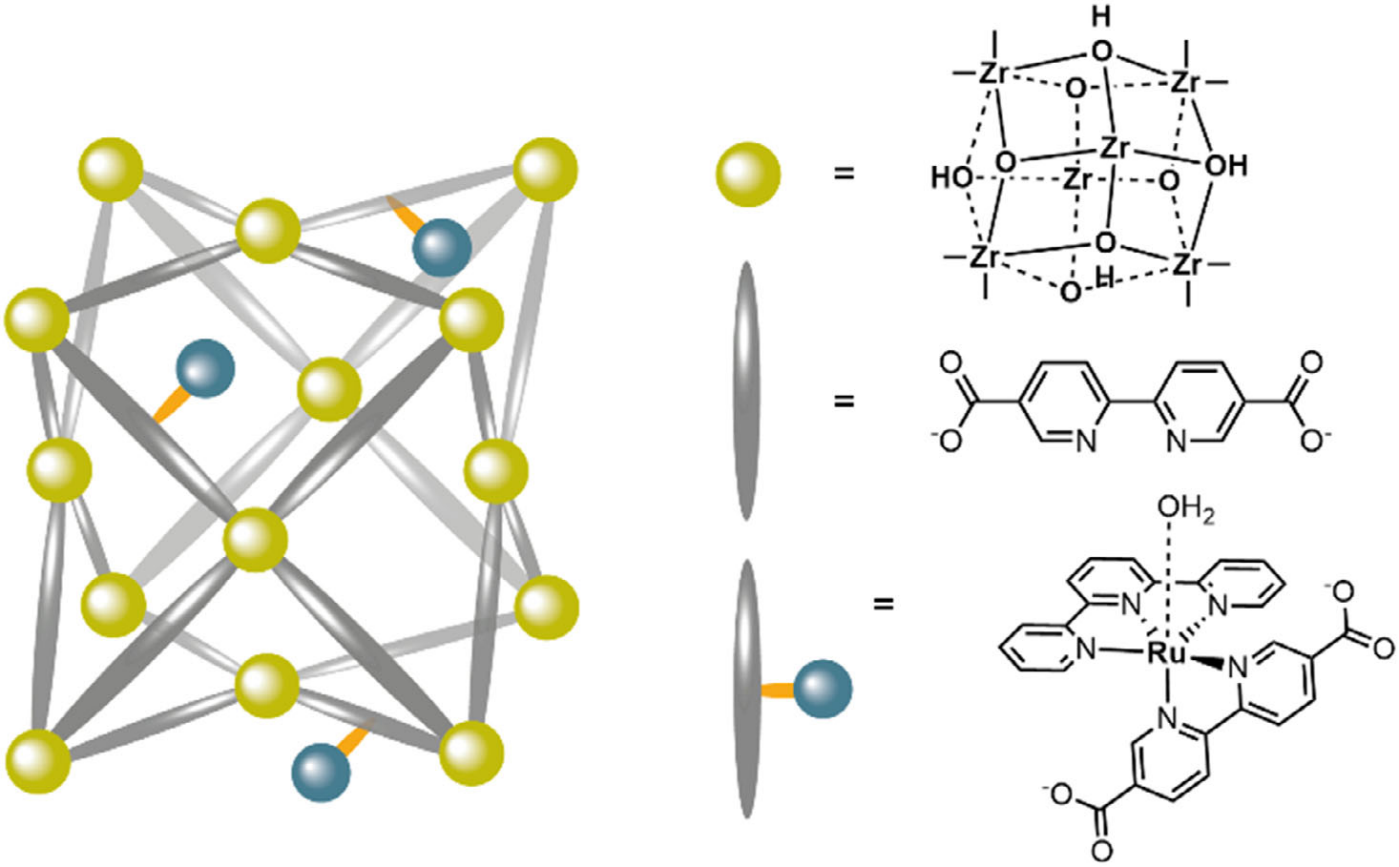
Schematic presentation of the **UiO|[RuOH2]|FTO** MOF.

The first MOF‐WOC was obtained by incorporation of the [Ru(tpy)(dcbpy)OH_2_]^3+^ (dcbpy = dicarboxylic acid bipyridine) WOCs by post synthetic ligand exchange into an UiO‐67 thin film grown onto FTO, to form **UiO|[RuOH**_**2**_ **]|FTO** (**Figure** **8**).^[^ ^79^ ^]^ The MOF film exhibited water oxidation activity with a faradaic efficiency of 82% for O_2_. The water oxidation onset potential is 1.4 V vs. Ag/AgCl at pH 6 and 1.1 V vs. Ag/AgCl at pH 8. Such a pH‐dependent onset is expected for the WNA mechanism as deprotonation of water occurs simultaneously with nucleophilic attack on the M=O species. Approximately 6% of the UiO‐67 linkers was displaced by a ruthenium functionalized linker, resulting in a number of catalytic sites immobilized on the electrode that is two orders of magnitude higher compared to the theoretical maximum number of catalysts that one would have in a monolayer. The reversible redox couples ascribed to the molecular Ru‐complex were observed both before and after catalysis, indicating that the catalyst is stable during these experiments. However, relatively low catalytic current densities were obtained, which was ascribed to the nonconductive nature of UiO‐67, and a poor charge transport that is fully dependent on electron hopping between the diffuse Ru centers present on 6% of the MOF linkers. Due to the low activity of the catalytic systems, and due to the equivalent homogeneous systems being particularly studied in presence of sacrificial oxidants, it is difficult to estimate to which extend embedding of the ruthenium sites within the UiO‐67 MOF leads to a more competent catalytic species.^[^ ^70^, ^76^ ^]^

### 4.2 Ru(tda)(py(PhCOOH)_2_)_2_

A Ru‐catalyst with a bipyridine dicarboxylic acid (bda) ligand was reported by the group of Sun to perform water oxidation with a very high activity.^[^ ^80^ ^]^ The catalyst forms dimers of two Ru^IV^—O species to form the O—O bond via the I2M mechanism. Three years later, Llobet and coworkers reported [Ru(tda)(py)_2_], (tda = tripyrdine dicarboxylic acid) with an even higher activity of TOF = 50 000 s^−1^ at pH 10 and 7700 s^−1^ at pH 7.^[^ ^81^ ^]^ Interestingly, this catalyst forms the O—O bond through the WNA mechanism. Anchoring the catalyst to multiwalled carbon nanotubes (MWCNTs) by functionalizing the pyridine ligand with a pyrene moiety, that binds to the MWCNTs by π‐π interactions, resulted in a highly stable catalyst with a TON > 10^7^.^[^ ^82^ ^]^

**Figure 9:**
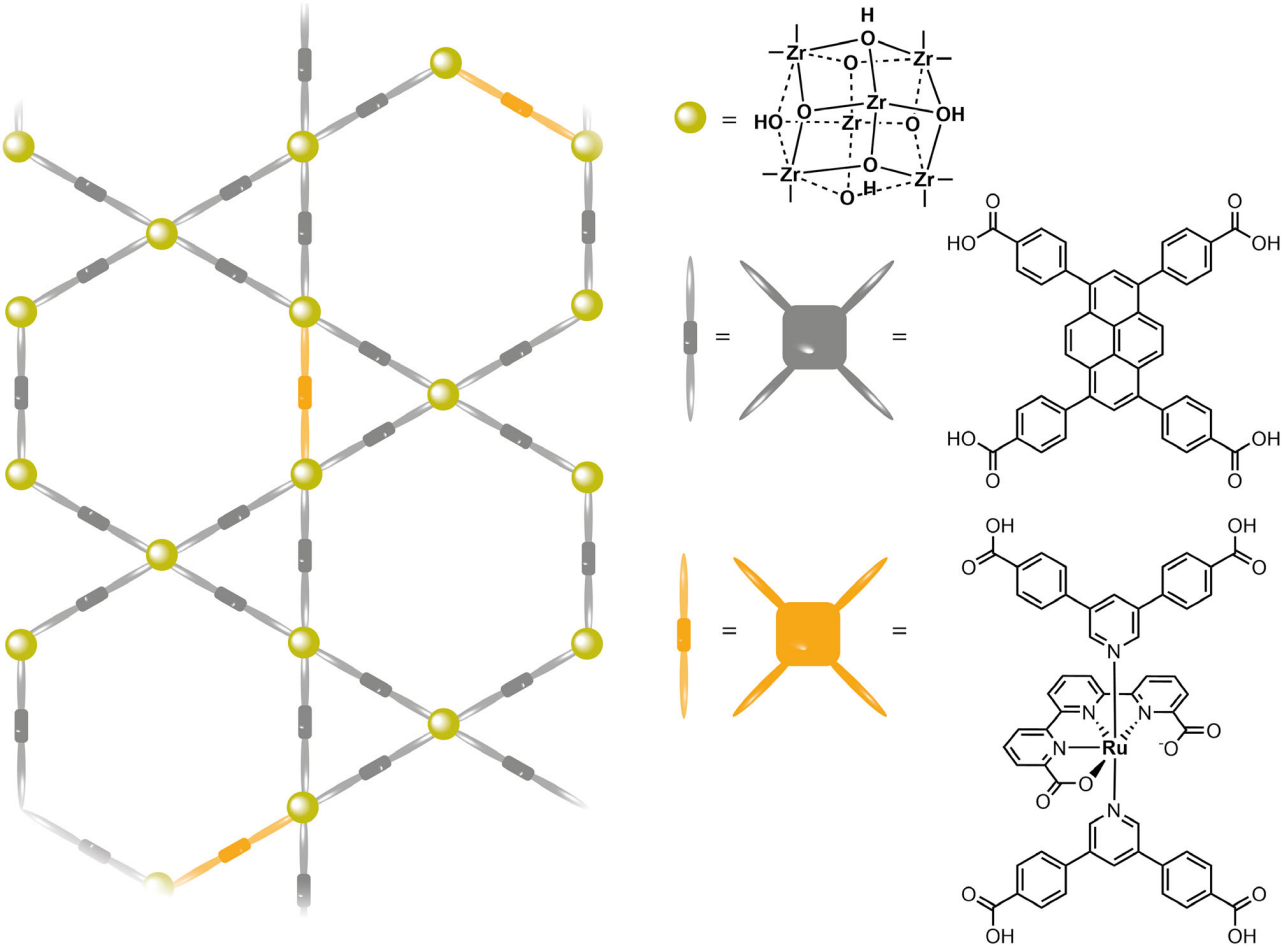
Schematic presentation of the mixed linker **NU1000|[Ru(tda)(py(PhCOOH)2)2]** MOF.

A NU1000‐type mixed linker MOF, with [Ru(tda)(py(PhCOOH)_2_)_2_] WOCs replacing some of the TBAPy linkers (**NU1000|[Ru(tda)(py(PhCOOH)**_**2**_ **)**_**2**_ **],** **Figure** **9**), was developed.^[^ ^30^ ^]^ The MOF was synthesized by mixed‐linker solvothermal synthesis with up to 30% of Ru‐linker, resulting in a maximum incorporation of almost 0.34 Ru linkers per node. The [Ru(tda)(py(PhCOOH)_2_)_2_] catalyst can be activated by CPE at 1.4 V vs. NHE for 40 min, after which it oxidizes water at a potential of 1.3 V vs. NHE at neutral pH. As TBAPy is also oxidized at 1.3 V vs. NHE, electrons are transported from the Ru‐catalyst, through the TBAPy linker and to the electrode when this potential is applied. In contrast to the **UiO‐[RuOH**_**2**_ **]|FTO** MOF based on the inert UiO‐67 framework, the TBAPy linkers in the **NU1000|[Ru(tda)(py(PhCOOH)**_**2**_)_**2**_] MOF are involved in charge transport from electrode to catalyst. The MOF suspended on MWCNTs shows water oxidation activity with a faradaic efficiency for O_2_ of 37%. The FE is relatively low due to the oxidation of the TABPy linkers. The authors argue that better matching of the redox potentials of the TABPy linker and the Ru‐catalyst would result in a larger proportion of catalytic sites engaging in catalysis. Since the oxidation of TBAPy occurs first, an initial period of CPE to oxidize all linkers followed by measuring oxygen content may lead to a possible underestimation of the FE. The catalytic activity of the MOF could not be directly compared to the catalyst in solution due to solubility issues. As an alternative, the [Ru(tda)(py(PhCOOH)_2_)_2_] catalyst suspended on MWCNTs was dropcasted for direct comparison to the MOF. Both the MOF and the catalyst suspended on MWCNTs showed the Ru^III/II^ and Ru^IV/III^ redox couples at similar potentials after activation by CPE at 1.2 V vs. NHE. The MWCNTs show a catalytic peak in CV starting at 1.4 V vs. NHE. The study by Llobet et al mentioned before reported TON > 10^7^, indicating the highly stable nature of this catalyst immobilized on MWCNTs.^[^ ^82^ ^]^ The MOF does not show a similar wave in CV but Clark electrode measurements during CPE at 1.3 V vs. NHE showed a stable increase of formed oxygen for 80 min.

### 4.3 Fe(salen)

The Schiff base complex Fe(salen) has been reported as a catalyst for various reactions in organic solvents such as oxidation of sulfides, polymerization of ethylene and propene, and oxidation of hydrocarbons in the presence of hypochlorite.^[^ ^83^, ^84^, ^85^ ^]^ No Fe(salen) catalysts have been reported where the molecular complex is the true catalyst for the oxygen evolution, but it has been reported as a pre‐catalyst for FeCo‐hydroxide with water oxidation activity.^[^ ^86^ ^]^ In order to stabilize the Fe(Salen) catalyst and prevent metaloxide formation, Fe(salen) has been incorporated into mesoporous silica,^[^ ^87^ ^]^ while two different Fe(salen)‐based MOFs were employed for the oxidation of sulfides.^[^ ^88^, ^89^ ^]^

**Figure 10:**
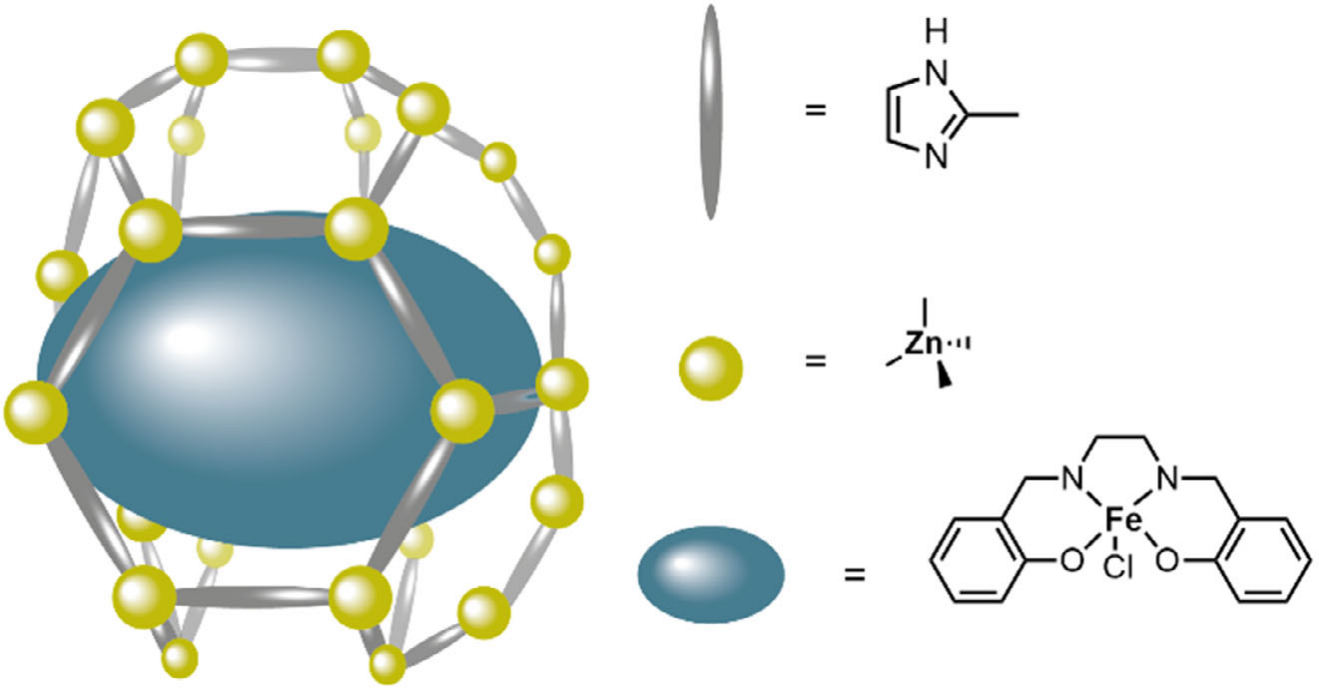
Schematic presentation of incorporation of Fe‐salen into the zeolitic framework ZIF‐8.

[Fe^III^‐(salen)(H_2_O)]^+^ and SiW_12_O_40_ clusters were incorporated into the Zn^2+^‐based MOF ZIF‐8 (**Figure** **10**) to form the highly stable **FSWZ‐8** MOF composite that is active for the oxygen evolution reaction.^[^ ^29^ ^]^ SiW_12_O_40_ is a polyoxometalate (POM), which is a well‐known electron shuttle (E^0^ (POM/POM^−^) = 0.054 V vs. NHE).^[^ ^90^ ^]^ The composite shows high activity, with FE = 95% and an onset of 1.1 V vs. NHE at neutral pH. A large catalytic current was observed with a peak current of 4 mA cm^−2^, compared to ZIF‐8 (no catalytic current) and ZIF‐8 with only [Fe^III^‐(salen)(H_2_O)]^+^ incorporated (**FSZ‐8,** 2.0 mA cm^−2^). The homogeneous [Fe^III^(salen)(H_2_O)]^+^ showed a peak catalytic current of 4 mA cm^−2^ that quickly decayed over consecutive scans. A high stability for the FSWZ‐8 MOF composite was demonstrated by 500 CV cycles and CPE 1.2 V vs. NHE for 6 h, in which the observed current did not change over time. A difference in UV‐vis, FTIR, and Raman spectra of the **FSWZ‐8** material compared to [Fe^III^‐(salen)(H_2_O)]^+^ indicated the presence of a different species in the MOF. A CV of [Fe^III^‐(salen)(H_2_O)]^+^ in homogeneous solution shows a complete disappearance of all catalytic activity over 10 cycles at 1.1 V vs. NHE. Since metal oxides can easily be formed under oxidative conditions, the instability observed in CV of [Fe^III^‐(salen)(H_2_O)]^+^ was explained by formation of FeOx during catalysis.^[^ ^91^ ^]^ Fe^IV^=O is the active oxidant in Fe‐salen catalysts and application of a high potential ensures this high oxidation state species to be constantly present in a high concentration, which may lead to degradation and formation of iron oxide.^[^ ^83^ ^]^ In order to evaluate if FeOx species are formed in situ during OER activity of **FSWZ‐8**, control experiments with three species of FeOx were formed and investigated by CV and Tafel analysis under the same conditions. In the first control, iron nanoparticles were deposited in ZIF‐8 by annealing the FSWZ‐8 MOF at 400 °C in presence of air to form **FeOx**_**400**_ **Z‐8**. In the second sample, FeOx was deposited on a carbon cloth (CC) electrode to form **FeO**_*x*_ **‐CC,** and in the third sample, iron oxide nanoparticles on ZIF‐8‐coated carbon cloth electrode (labeled as **FeO**_*x*_ **‐Z8**) were prepared through electrodeposition by applying high anodic potential in an aqueous solution of FeCl_3_ (1 mM). CV experiments of these controls show that these FeOx species had no OER activity at the potential at which **FSWZ‐8** operates under the applied reaction conditions. It was therefore concluded that the OER mediated by **FSWZ‐8** does not involve FeOx species. The authors attribute the stabilization of Fe‐salen in the MOF to its confinement within the MOF and the increased activity to effective charge transport involving the POM. Incorporation of the redox active POM with oxidation potential that is capable of oxidizing the Fe‐sites lowers the overall charge transfer resistance, via a similar effect as has been described for the **NU1000|CoPC** MOF in CO_2_RR.

## 5 Challenges

A handful of reports have shown interesting possibilities when incorporating a molecular electrocatalyst into a MOF; however, the field is still quite new and several major challenges remain. Some of these challenges are issues that many scientists working on metal–organic frameworks encounter, while others are topics that are not frequently discussed in the field but should be taken into account.

To the first category belong the issues relating to electron transfer in MOFs. The limiting factor for the catalytic reaction can be found in 1) slow electron transport, 2) slow mass transport of reactants and products, or 3) a low activity of the catalytic site itself, either caused by the confinement effects or an unfavorable electronic structure when integrated within the MOF. Electron transfer in MOFs can be diffusional charge transfer through π‐π stacking of aromatic moieties,^[^ ^92^, ^93^ ^]^ through‐bond electron transfer^[^ ^94^, ^95^ ^]^ or cation‐coupled electron hopping between redox active guests or linkers.^[^ ^96^, ^97^, ^98^ ^]^ When electrons are transferred by π‐π stacking of aromatic moieties, delocalized electrons are shared between the stacked aromatic rings. Dinca and coworkers showed Ga, Ni, and Co MOFs with the 2,3,6,7,10,11‐hexahydroxytriphenylene (H_6_HOTP) linker exhibited similar conductivities of around 3 × 10^−3^ S cm^−1^.^[^ ^92^ ^]^ This form of charge transport requires close proximity of linkers in the same plane and has a conductivity of typically 10^−4^ S cm^−1^. Through‐bond electron transfer is a form of intervalence charge transport and therefore depends on orbital overlap. It is the fastest form of charge transport in MOFs with a conductivity of typically 1 S cm^−1^. In the Fe(tri)_2_(BF_4_)_*x*_ (tri^−^ = 1,2,3‐triazolate; *x* = 0.09, 0.22, and 0.33) MOF, electrons are transported by conjugation of the trizolate linker *π*‐orbitals and the Fe 3*d* orbitals.^[^ ^95^ ^]^ Cation‐coupled electron hopping depends on the distance between two electro‐active moieties as well as the diffusion of cations. Cation‐coupled electron hopping can be determined by Cottrell analysis of CPE measurements are typically reaches conductivities of 10^−10^–10^−12^ S cm^−1^. The discrepancy between the electronic conductivity by the through‐bond, π‐π stacking, and electron hopping mechanisms is notable and improving electron hopping rates is a major topic of investigation.^[^ ^96^ ^]^ Even though redox‐active MOFs can be used to enhance charge propagation, matching the potentials of the redox active sites within the MOF and catalyst is crucial while discrepancies in redox potential will lead to slow electron transfer processes. When the potential of the linker is more negative than the potential of the catalyst, either the linkers are not reduced and form a redox‐innocent framework, or the catalytic reaction can only be performed at a high overpotential, where also the linker is reduced. On the other hand, if the redox potential of the catalyst is much more negative than the potential of the linker, the linker will not be able to reduce the catalyst and will likely accumulate electrons without turning over. It is also important to note that charge propagation studies are often performed in DMF or other organic solvents while for catalytic purposes for a green energy economy, water is often the preferred solvent and charge propagation speed and mechanisms may vary with solvent polarity.^[^ ^96^, ^99^, ^100^ ^]^ Additionally, when charge transport in is coupled to ion migration in aqueous electrolyte, it is not far‐fetched to assume that proton‐coupled electron transfer is involved. In this case, electron transfer rates may be directly dependent on the electrolyte pH and the pKa values of nodes and linkers.

Furthermore, an appropriate electronic connection between the support and the MOF is expected to be essential for good electron transport as well. The method in which the MOF is immobilized (e.g., as a carbon black–Nafion ink, or directly grown on FTO), but also the potential defects that accompany anchoring of the MOFs, are likely important factors to consider.

A second challenge is the stability of MOFs. Often, changes in morphology or complete delamination of a MOF thin film are observed during catalysis.^[^ ^60^, ^79^, ^101^ ^]^ For example, the on FTO grown thin‐film **Re‐SURMOF** for CO_2_ reduction completely delaminated, and SEM images before and after electrolysis showed morphology of Cu‐adeninato MOF (Cu‐ade‐MOF) changed completely after CO_2_ reduction electrolysis. A strategy for improving stability of MOFs grown directly onto FTO substrates would be to create a monolayer of the node material, such as ZnO or ZrO, by atomic layer deposition (ALD) onto the FTO substrate, which then can be used as a platform to grown more stable MOF**|**FTO structures.^[^ ^102^, ^103^ ^]^ This strategy may avoid instable bonds between linkers and FTO, resulting in MOF delamination. Mechanisms of catalyst degradation are rarely investigated, making it difficult to know whether degradation is triggered by redox reactions occurring at high potentials, or by chemical reactions involving solvents or products formed. A good stability toward a wide pH range is essential for MOFs for reactions such as water oxidation, hydrogen evolution, and CO_2_ reduction. These reactions may result in significant pH gradients in the MOF pores, particularly if proton transport is retarded. As improving the stability of the homogeneous catalyst is one of the main motivations for incorporating it into an MOF, it is imperative to investigate these mechanisms of MOF degradation. Given that a local pH swing to alkaline and acidic conditions is expected for reduction and oxidation reactions, respectively, we recommend taking into account such pH swings when selecting an appropriate MOF to accommodate an electrocatalyst.

At present mass transport in MOFs is still poorly understood. For example, the effect of rotating an electrode on mass transport inside of the framework is not investigated. In electrochemical setups with rotating electrodes, as well as GDEs and flow cells, mass transport to the electrode is increased, and therefore also to the MOF–electrolyte interface, but whether and to what extent mass transport within pores is also altered remains unclear to this date. Additionally, the effect of partially blocked pores because of incorporated catalysts, interpenetrated MOF structures^[^ ^104^ ^]^ or MOF defects on the MOF porosity and therefore mass transport within the MOF has not been investigated. Morris and coworkers have shown in organic solvent that ion diffusion is rate limiting in charge transport rather than the electron hopping rates, and that charge transfer through MOFs increases with increasing pore sizes.^[^ ^105^ ^]^ Related to this, the exact location where catalysis occurs is often not clear, particularly when only a small fraction of sites is catalytically active. Ott and coworkers described with mathematical reaction‐diffusion models how a catalytic current can be limited by mass‐ or charge transport.^[^ ^106^ ^]^ They show that when mass transport is limiting, the reactants will be consumed by catalysts at the MOF–electrolyte interface and catalysts deeper into the framework will not be catalytically active. When electron transport is limiting, catalysts far from the electrode surface will not be activated and catalysis will take place close to the electrode–MOF interface. They also show that the optimal thickness of the catalytic MOF depends on the intrinsic activity of the catalyst: when a relatively slow catalyst is used, a thicker MOF film, containing more catalytic sites, can be used as there is enough time for the substrate to diffuse through the pores to catalysts deeper in the framework. When a catalyst is very active, the optimal MOF film thickness is smaller as diffusion will always be slower than the reaction. These models can be used to design MOFs with optimized pore structure and film thickness for substrate and charge diffusion.

A challenge that is usually overlooked but that we consider highly important to discuss is that still very little is known about the effect that confinement within an MOF has on a molecular catalyst. The catalytic mechanisms of the homogeneous catalyst are often only studied in homogeneous solution, and not in the MOF. It is assumed that the mechanism by which the catalyst operates, and the catalytic species present, do not change when a catalyst is confined in a framework. This is not necessarily correct. Which reaction pathway is dominant often is very much dependent on the reactant concentration, and thus on mass transport through the MOF. And as illustrated earlier, particularly local pH swings are expected to play a significant role in MOF electrochemistry. These severe local pH effects can in turn have many effects on catalytic mechanisms and mechanisms of instability. Rarely the catalytic results of MOF embedded catalysts are directly compared to the catalytic results obtained for the homogeneous catalyst under identical conditions. This makes it difficult to judge the effectiveness of confinement of the catalyst and to truly identify the potential of the field of electrocatalysis employing MOFs. We therefore emphasize that detailed mechanistic studies carried out within the confinement of MOFs are indispensable to bring the field of MOF electrocatalysis to a more developed stage.

## 6 Conclusions and Recommendations

The confinement of molecular catalysts for reactions relevant to the energy transition in metal–organic frameworks have been shown to lead to significant improvements in selectivity, long‐term activity, and catalyst stability. This is particularly due to the exclusion of bimolecular pathways thereby shutting down bimolecular catalyst degradation and other side phenomena. Although initial strategies to improve electron transfer throughout MOFs have been described, major knowledge gaps in how electron transport occur precisely during electrocatalysis, how mass transport of reactants occurs during and limits electrocatalysis, and how local pH swings can be avoided still exist. It is expected that these research questions remain an important area of research within the next decade. Initial hints regarding the effect of confinement of catalyst within an MOF have been summarized. Yet to fully utilize catalysts incorporated in MOFs, to develop the electrocatalysts that are needed for a fully sustainable society, significantly more systematic research will be required.

## Conflict of Interest

The authors declare no conflict of interest.
